# Effects of angular gain transformations between movement and visual feedback on coordination performance in unimanual circling

**DOI:** 10.3389/fpsyg.2014.00152

**Published:** 2014-03-05

**Authors:** Martina Rieger, Sandra Dietrich, Wolfgang Prinz

**Affiliations:** ^1^Department of Psychology, Max Planck Institute for Human Cognitive and Brain SciencesLeipzig, Germany; ^2^Department for Medical Sciences and Management, Institute for Psychology, University for Health Sciences, Medical Informatics and TechnologyHall in Tirol, Austria; ^3^Department of Education, Leipzig UniversityLeipzig, Germany

**Keywords:** unimanual coordination, visuo-motor transformation, gain transformation, sensorimotor integration, tool transformation, circling, synchronization

## Abstract

Tool actions are characterized by a transformation (of spatio-temporal and/or force-related characteristics) between movements and their resulting consequences in the environment. This transformation has to be taken into account, when planning and executing movements and its existence may affect performance. In the present study we investigated how angular gain transformations between movement and visual feedback during circling movements affect coordination performance. Participants coordinated the visual feedback (feedback dot) with a continuously circling stimulus (stimulus dot) on a computer screen in order to produce mirror symmetric trajectories of them. The movement angle was multiplied by a gain factor (0.5–2; nine levels) before it was presented on the screen. Thus, the angular gain transformations changed the spatio-temporal relationship between the movement and its feedback in visual space, and resulted in a non-constant mapping of movement to feedback positions. Coordination performance was best with gain = 1. With high gains the feedback dot was in lead of the stimulus dot, with small gains it lagged behind. Anchoring (reduced movement variability) occurred when the two trajectories were close to each other. Awareness of the transformation depended on the deviation of the gain from 1. In conclusion, the size of an angular gain transformation as well as its mere presence influence performance in a situation in which the mapping of movement positions to visual feedback positions is not constant. When designing machines or tools that involve transformations between movements and their external consequences, one should be aware that the mere presence of angular gains may result in performance decrements and that there can be flaws in the representation of the transformation.

## INTRODUCTION

Movements of the limbs are limited by the speed and the distance they can cover without moving the whole body at the same time. Tools, however, allow us to overcome motor system limitations. By using tools, we can reach distances out of bodily reach or achieve movement effects in the environment which are faster or slower than our actual movements. Tool use requires that an adjustment to some type of transformation between motor activity and resulting consequences in external space takes place. The transformation can be kinematic (i.e., refers to the relationship between the spatio-temporal characteristics of limb movement and the associated spatio-temporal characteristics of the tool movement) and/or dynamic (i.e., refers to the relationship between the forces the limb exerts and the forces that a tool exerts on the environment; Massen and Rieger, 2012). Kinematic transformations consist of two aspects (see Bedford, 1994). First, the consequences in external space happen in a different location than the actual motor activity. For example, when using a computer mouse motor activity takes place on a mouse-pad but the resulting consequences happen on a computer screen. Second, the term transformation indicates that the mapping between motor activity and consequences in external space is not 1:1. When using a computer mouse or a touchpad the cursor on the screen covers a larger distance than the actual movement (correspondingly, the speed of the feedback is faster than the actual movement, gain larger than 1). When driving a car, turning the steering wheel by 90° does not result in the wheels also turning by 90°, but less (gain smaller than 1). Thus, use of tools implies that a transformation has to be taken into account when planning and executing movements. The transformation itself seems to be an important part of the cognitive representation of tool-use actions (Massen and Prinz, 2007).

In the present study we were interested in gain transformations, a specific way to vary the mapping between motor activity and its consequences in external space. A transformation of gain means that the resulting consequences in external space are larger or smaller than the actual movement, as it is the case when using a computer mouse or turning a steering wheel. Gain transformations are generally thought to be easy to adapt to (Bedford, 1994; Bock and Burghoff, 1997; Seidler et al., 2001; Rieger et al., 2005), For example, drawing three strokes after a gain change is introduced is sufficient for adaptation (Rieger et al., 2005).

Gain transformations also influence movement difficulty as described by Fitts’ Law (Fitts, 1954). Movements are more difficult (i.e., movement time is higher, they are performed less accurately) with higher gains than with lower gains (Rosenbaum and Gregory, 2002; Rieger et al., 2005; Mohler et al., 2007; Sutter et al., 2008). For instance, Mohler et al. (2007) asked participants to walk on a treadmill. They received visual input of half, the same, or twice the speed of actual walking. Preferred walking speed was lower with doubled visual speed and higher with halved visual speed compared to when visual and walking speed were the same. Similar results have been obtained with hand movements (Rosenbaum and Gregory, 2002; Rieger et al., 2005; Sutter et al., 2008). Again, movements are more difficult with higher gains, resulting in a deterioration in endpoint accuracy when movement frequency is given (Rosenbaum and Gregory, 2002), or in slower movements when participants are free to choose their movement speed but are instructed to adhere to spatial accuracy requirements (Rieger et al., 2005; Sutter et al., 2008). Presumably, those adjustments reflect that the cognitive system tries to maximize the predictability of the perceived trajectory in external space.

Most of the previous studies have investigated the influence of different gains in movements along a straight line (along the medial or saggital axis). In contrast, in the present study we investigated transformations scaling gain in circling movements. Such a transformation is for example present when using a hand driven spinning wheel. A hand driven spinning wheel requires that one hand rotates a drive wheel (usually the bigger wheel, which is often rotated by a handle) which turns the smaller spindle assembly, with the spindle turning several times for every turn of the drive wheel. Circling movements differ from movements in a straight line when an angular gain unequal to 1 between the movement and its feedback is introduced. Whereas the mapping of positions on the movement trajectory to positions on the visual trajectory is constant in movements on a straight line, this is not the case in circular movements. Rather, an angular gain unequal to 1 in a circular movement results in a constant change of the mapping of positions on the movement trajectory to positions on the visual trajectory, even though the gain itself remains constant. As an example, imagine that the starting position of the movement trajectory and the starting position of the visual feedback trajectory are both on the right side of a circle. If a gain of 1.5 is introduced, after moving one circle in movement space (the hand is again on the right side), 1.5 circles in visual space have been covered, and now the visual feedback is on the left side of the circle. After another circle in movement space, hand and visual feedback are both on the right side again: in movement space, two circles have occurred, in visual space three circles have occurred.

Circling movements have often been investigated in bimanual coordination studies (e.g., Swinnen et al., 1997). Research on bimanual coordination has demonstrated that people are more accurate and consistent if they execute bilateral mirror symmetric movements (movements in which the hands move toward and away from the body midline at the same time, e.g., moving one hand clockwise and the other hand counterclockwise) than when they perform any other type of movement pattern (e.g., moving both hands clockwise, Swinnen et al., 1997). Transformed visual feedback has been used to study the relevance of motor constraints/motor related feedback (kinesthesis and proprioception) and perceptual-cognitive constraints/visual feedback for coordination performance. For instance, visual feedback of a circling movement has been shifted 180° (Tomatsu and Ohtsuki, 2005), or transformed to result in an easily perceivable pattern (mirror-symmetric, Mechsner et al., 2001, Lissajous displays, Kovacs et al., 2010a,b) such that participants are able to perform complicated or awkward bimanual movement patterns (such as 4:3, Mechsner et al., 2001), which are otherwise impossible or very difficult to perform. These studies indicate that visual processes play an important role for bimanual coordination (see also Bogaerts et al., 2003; Mechsner, 2004). The perceptual ease of horizontally aligned symmetry information is also illustrated by perceptual studies: it is easier to judge images which are mirrored along a horizontal axis than images which are mirrored along a vertical axis (Quinlan, 2002). We therefore decided to instruct participants to coordinate transformed movement feedback with a stimulus in a way that a symmetric pattern emerges in visual space, which should be perceptually easy.

Coupling phenomena found in bimanual coordination tasks seem to persist in unimanual coordination, i.e., when coordination occurs between a single limb and a computer display (e.g., Wimmers et al., 1992; Buekers et al., 2000). In unimanual coordination there is no second limb with which movements need to be coordinated, but rather a coordinative stimulus/event. Since there can be no constraints on the motor level related to bimanual coordination (only one hand is moving), unimanual coordination has to follow the perceptual characteristics of the movement feedback, which can be either visual and/or proprioceptive/kinesthetic. Studies indicate that visual feedback dominates in many situations of unimanual coordination (Buekers et al., 2000; Roerdink et al., 2005; Dietrich et al., 2012). However, the states of the limb, and the perception of those states, must also be taken into account (Wilson et al., 2005a,b). Further, it depends on the type of task whether visual or kinesthetic/proprioceptive information is most beneficial (Alaerts et al., 2007). Similar to the present task, Dietrich et al. (2012) asked participants to perform a unimanual coordination task that required participants to coordinate the visual feedback of hand movements with a circling stimulus. To dissociate movements and the associated proprioceptive/kinesthetic feedback from visual movement feedback, participants performed the task under regular and transformed visual feedback (180° angular shift). Results indicated that coordination mainly occurs in visual space (similar data patterns with regular and transformed feedback), but subtle effects of coordination in movement space were also observed. Further, the presence of a transformation affected performance negatively. Thus, if movement and its feedback do not correspond, performance may suffer. However, the transformation in Dietrich et al. (2012) did not consist of a gain transformation, but rather a constant shift of the feedback relative to the hand. Müsseler and Sutter (2009) also investigated transformed circular movements. Participants drew circles on a display while the hand movements followed either vertical or horizontal ellipses. Even though a gain transformation was involved to achieve this feedback (either in the *x*- or *y*-axis), the mapping of movement positions to feedback positions was constant, similar to when gain transformations are introduced in movements on a straight line. In contrast to those studies, in the present study gain transformations were introduced in such a way that the mapping of movement positions to feedback positions was not constant. The effect of such a transformation on performance as well as on awareness of the transformation is largely unknown.

In the present study, we used a unimanual coordination task in order to investigate how the perceptual-motor system deals with angular gain transformations resulting in a non-constant mapping of movement positions to feedback positions in circling. Participants were asked to coordinate a feedback dot (produced by the participants’ movement and presented on the right side of a screen) with a continuously circling stimulus dot (presented on the left side of the screen), in order to produce mirror symmetric circular movements of the two dots on the screen. The movement angle of the hand was multiplied by a gain factor before being presented on the screen: we used 4 gains smaller than 1, a gain of one, and 4 gains larger than 1. This allowed us not only to compare transformed vs. regular conditions (e.g., Mechsner et al., 2001; Roerdink et al., 2005; Dietrich et al., 2012), but also to study the impact of transformation magnitude on coordination performance.

If only perceptual characteristics in visual space are important for unimanual coordination, the different gains between hand movement and its feedback should have no effect on performance, as the pattern participants were asked to produce in visual space was always the same. Thus, *accuracy* of performance, i.e., the time participants spend in the instructed visual pattern, should be equal for different gains. If movement speed, or some biomechanical variable related to movement speed, is important for coordination performance, performance should decline the smaller the gain, because smaller gains imply more distance has to be covered by the hand movement to produce the desired distance on the screen. Therefore movements have to be faster. However, if it matters that a transformation is introduced between movement and its feedback, the best performance should be observed at a gain of 1 and performance should be worse at both, gains smaller and gains larger than 1. If performance is worse in gains unequal to 1, we were further interested in whether the magnitude of the transformation matters for performance. On the one hand, one could expect that all gains which are not equal to 1 are performed equally well (or bad), because they all imply a constant change in the mapping of hand position to feedback position. On the other hand, the mapping change is more drastic in gains which show a larger deviation from 1 than in gains that show a smaller deviation. Thus, performance may vary gradually.

We further varied the speed of the stimulus dot in three levels, because previous studies have shown that coordination performance deteriorates when movement and/or feedback speed increases (Kelso, 1984; Haken et al., 1985; Heuer, 1993; Byblow et al., 1995; Carson et al., 1997; Roerdink et al., 2005), especially under transformation conditions (e.g., Salter et al., 2004; Alaerts et al., 2007; Dietrich et al., 2012). We therefore expected to find deterioration in performance with increasing speed.

In addition to the accuracy of performance, we were interested in *how* participants perform the task. First, we were interested in whether participants’ movement feedback is on the ideal position as instructed (in perfect mirror symmetry), or whether it systematically lags behind or is advance of (leads) that position. We assumed that the feedback dot would be in advance of the stimulus dot, as the movements were performed with the right (dominant) hand and the feedback was presented on the right side of the screen. In bimanual coordination the dominant hand usually shows a slight lead over the non-dominant hand when coordinating symmetrical movements (Treffner and Turvey, 1995), an effect which seems to be due to attention rather than motoric factors, because the lead of the dominant hand disappears when attention is directed to the non-dominant hand (Amazeen et al., 1997). However, this lead might be affected by the gain transformation, because gain transformations may evoke subjective feelings of feedback being slow or fast.

The second way to investigate how participants perform the task was to analyze whether they show anchoring, that is, a reduced variability at specific locations on the trajectories (Roerdink et al., 2008). Regions at which anchoring occurs are often located at or near movement reversals or maximal excursions (e.g., Beek, 1989; Kelso and Jeka, 1992; Byblow et al., 1995; Fink et al., 2000), that is regions in which critical task-specific information is available for organizing cyclical movements (Beek, 1989; Kelso and Jeka, 1992). In addition to reducing kinematic variability at/around movement transition points anchoring stabilizes entire movement cycles (Roerdink et al., 2008). Anchoring has therefore often been regarded as a motoric phenomenon. However, Roerdink et al. (2005) found support for visual as well as motoric contributions to anchoring. Furthermore, Roerdink et al. (2008) found that anchoring in visual space and in movement space were independent from each other. Usually, anchoring is studied in reference to externally generated events like a metronome (e.g., Fink et al., 2000), or in relation to self-generated events like movement reversals (Roerdink et al., 2008), ball release in juggling (Beek, 1989), or feedback tones in tapping (Keller and Repp, 2008), all of which provide discrete information which can be used for anchoring. Such information was not available in our task. We therefore assumed that anchoring would occur in a visually salient location, that is, when the two dots are closest together in the middle of the screen. Due to the non-constant mapping of movement positions and feedback positions and correspondingly between movement positions and stimulus positions, such a position is difficult to conceive in movement space, we therefore investigated anchoring only in visual space.

We were further interested in whether awareness of the transformation depends on the magnitude of the transformation or whether a mismatch between movement and feedback position (i.e., any transformation) is sufficient to detect the transformation. Previous studies indicate that participants are not very good in knowing their actual hand positions when transformations between movements and their feedback are introduced and that the magnitude of a perturbation plays an important role for detecting it (Fourneret and Jeannerod, 1998; Knoblich and Kircher, 2004; Sutter et al., 2008; Müsseler and Sutter, 2009). This low awareness of oneüs own hand movement seems to stem from characteristics of the tactile and proprioceptive systems as well as insufficient spatial reconstruction of this information in memory ((Müsseler and Sutter, 2009). Based on the previous studies, one can expect that the detection of the transformation depends on the magnitude of the gain. However, even with small gain transformations positions in movement and visual space eventually become very discrepant. For instance, with a gain of 1.2, 2.5 circles in movement space result in three circles in visual space, and hand position and feedback positions are thus on opposite sides of the circle. Thus, the mere presence of a transformation may be important for its detection in the present task, but not its size.

## MATERIALS AND METHODS

### PARTICIPANTS

Fourteen adults (eight female and six male, aged 20–28 years, mean = 24.6 years, SD = 2.2 years) took part in the experiment. Originally two more participants participated, but they were excluded from data analysis because they had difficulties performing the task. All participants were right-handed according to the Edinburgh Handedness Inventory (Oldfield, 1971) and reported normal or corrected-to-normal vision. They were paid seven Euros/hour to participate in a single session Participants gave informed consent. The study was conducted in accordance with the Declaration of Helsinki and was approved by the local ethics committee.

### APPARATUS AND STIMULI

The experiment was programmed using the C-language in a Microsoft DOS environment. Movements were recorded using a Wacom UD A3 writing pad (resolution: 500 pixels per centimeter, sampling rate 100 Hz), which was connected to the computer via a serial port. The serial port was open all the time and as soon as a new data sample was available this sample was further processed by the program. The writing pad was positioned on a desk horizontally in front of participants. Stimuli were presented on a 17^″^ screen (refresh rate: 75 Hz, resolution: 800×600 pixels, positioned vertically). The center of the screen was aligned with the midsagittal axis of the participantüs body and located behind and 15 cm higher than the writing pad. The background of the screen was black.

The stimulus was presented as a white dot (diameter = 0.43 cm, stimulus dot), moving clockwise on a circular trajectory (radius = 4.32 cm). A second white dot (feedback dot, radius 0.43 cm) was controlled by a stylus for the writing pad. The stylus was fixed inside a crank (radius 5 cm) that participants held, which could only be moved in circles. The crank was fixed below a wooden board (15 cm above the writing pad), which also served to shield the hand from view. The center of the circular trajectory of the hand was positioned 10 cm to the right of the body midline. The distance between the centers of the stimulus and feedback trajectories on the screen was 17.27 cm. Participants sat on a height-adjustable chair, which they could adjust to their comfort before the experiment started. Eye-screen distance was approximately 60 cm.

### PROCEDURE AND DESIGN

Participants were instructed to produce mirror symmetric movements of the dots on the screen: they were always asked to move their hand in counter-clockwise direction and to match the speed of the feedback dot to the speed of the stimulus dot (which always moved clockwise). The stimulus dot was presented in three different speeds; 0.8, 1, and 1.2 Hz (i.e., 0.8, 1.0, and 1.2 circles per second, respectively). The relation of the speed of the hand movement and the speed of the feedback dot was manipulated by introducing different gains.

**FIGURE 1:**
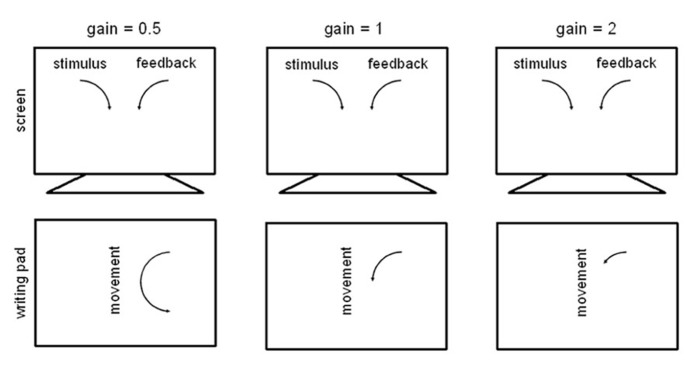
**Illustration of the gain manipulations.** Altogether nine different gains were conducted in the experiment: 0.5, 0.6, 0.75, 0.8, 1, 1.25, 1.3, 1.5, and 2. Note that the depiction of stimulus, feedback and movement are not scaled to actual size on the screen and on the writing pad.

The angle the hand moved between two measuring points (angular displacement) was multiplied by a gain factor between 0.5 and 2 before being displayed on the screen at the next refresh. The average delay until a data sample were presented on the screen was 7.67 ms, the maximum delay was 14.33 ms. This was due to the refresh rate of the screen and a maximum of 1 ms for data transmission and to perform the necessary calculations. There were nine different gains, 4 smaller than 1 (0.5, 0.6, 0.75, 0.8), requiring the hand movement to be faster than the movement of the feedback dot (MoFast gains), gain = 1, and 4 larger than 1 (1.25, 1.3, 1.5, 2), requiring the hand movement to be slower than the movement of the feedback dot (MoSlow gains). For an illustration see **Figure 1**.

The experiment started with a short trial in which participants were asked to turn the crank in order to check whether the writing pad worked properly and to allow participants to familiarize themselves with the apparatus. After that participants read the instructions and saw a demonstration of the mirror symmetric pattern they were later asked to produce. The demonstration consisted of two dots in the positions of the stimulus and feedback dots, moving clockwise and counter-clockwise, respectively. Participants were told that in the experiment the feedback dot would sometimes cover a larger or smaller angular distance than their hand and that they would occasionally be asked to indicate how likely they considered the presence of such a transformation in a preceding trial. They were also told that the speed of the stimulus dot increases during each trial. After that, the procedure was the same for every trial. Participants were instructed to hold their hand in the leftmost position at the beginning of a trial. They started trials themselves by pressing the space bar on a keyboard with their left hand. As soon as the space bar was pressed the stimulus dot appeared at the rightmost position of the stimulus trajectory and started moving. The stimulus dot increased its speed every 10 circles by 0.2 Hz (one trial thus consisted of all three speeds). Each trial lasted 30.83 s. Each gain was presented in one block for eight trials. After the sixth trial in each block participants were asked to rate whether a transformation was present in the last trial on a scale from 1 to 5 (1 = certainly not present; 2 = likely not present; 3 = undecided; 4 = likely present; 5 = certainly present), which was presented on the screen. Participants’ decision was recorded by the experimenter. The order of gains (i.e., the nine blocks) was randomized between participants. After five blocks there was a break of at least 3 min. It took participants between 1 h and 1 h 30 min to complete an experimental session, as they had the opportunity to take brakes for as long as they wished between trials.

### DATA ANALYSIS

Because we were interested in performance after participants have adjusted to a certain transformation and not in the process of adaptation, we excluded the first three trials of every block from analysis, as they were regarded as training trials. Further, we excluded the first three circles of every speed level, to allow time for adaptation to the new speed requirements. For each remaining data point we calculated the angular difference by subtracting the ideal position of the feedback from the actual position of the feedback. Because the shortest distance between the two points was used, the angular difference cannot be smaller than -180° or larger than 180°.

Based on the angular difference values we calculated the percentage of time participants spent in the instructed pattern [Instructed Mode (IM); angular differences between -45 and 45°] in order to assess the *accuracy* of coordination. The expected value (if performance is random) is 25%. In order to assess *how *the task was performed, we calculated the spatial Constant Error (CE), a signed value indicating the average angular difference between the ideal and the actual angle, which indicates whether participants are in lead of or lag behind the stimulus. We also calculated the temporal CE. The data patterns of the spatial and temporal CE were very similar (as they are related in our task). We therefore decided only to report the spatial CE.

Further, as an indicator of anchoring, we analyzed the spatial variable error (VE), the standard deviation of the CE, at four locations of the stimulus trajectory (east, south, west, and north, as in a compass card). Note that east in the stimulus trajectory meant that participants were supposed to be in the west of the feedback trajectory. To calculate VE, we defined windows of 30° around the respective points of interest. A window of 30° was chosen in order to (a) cover a relatively narrow area around the points of interest and (b) still have several measuring points even with higher speeds. Angular difference values within this window were averaged for each circle. Then the standard deviation across circles was calculated from those values. Thus, VE describes the variability of the movement position across circles in those areas. We also calculated the temporal VE, as it has been argued temporal and spatial aspects of anchoring should be separated (e.g., Roerdink et al., 2008). The data patterns of the spatial and temporal VE were very similar (again because they are related in our task). However, spatial VE increased with speed, whereas temporal VE decreased with speed. This is in accordance with studies showing that spatial variability is inversely related to movement time, whereas temporal variability is positively related to movement time (Schmidt et al., 1979). Since no additional information was gained from temporal VE, we only report the spatial VE.

Instructed Mode and CE were then analyzed using ANOVAs with the factors Gain (0.5, 0.6, 0.75, 0.8, 1, 1.25, 1.3, 1.5, and 2) and Visual Speed (0.8, 1, and 1.2 Hz). VE was analyzed with the additional factor location (east, south, west, north). *Post hoc* comparisons were conducted using *t*-tests. The ratings of the presence of a transformation were analyzed only with the factor gain using Friedman’s test, Wilcoxon signed-rank test were conducted as *post hoc* tests. The significance level for *post hoc* tests was corrected using the Holm–Sídák procedure, where appropriate exact, minimum (*p*min) and/or maximum (*p*max) *p*-values are reported.

## RESULTS

### ACCURACY OF PERFORMANCE: INSTRUCTED MODE

**FIGURE 2:**
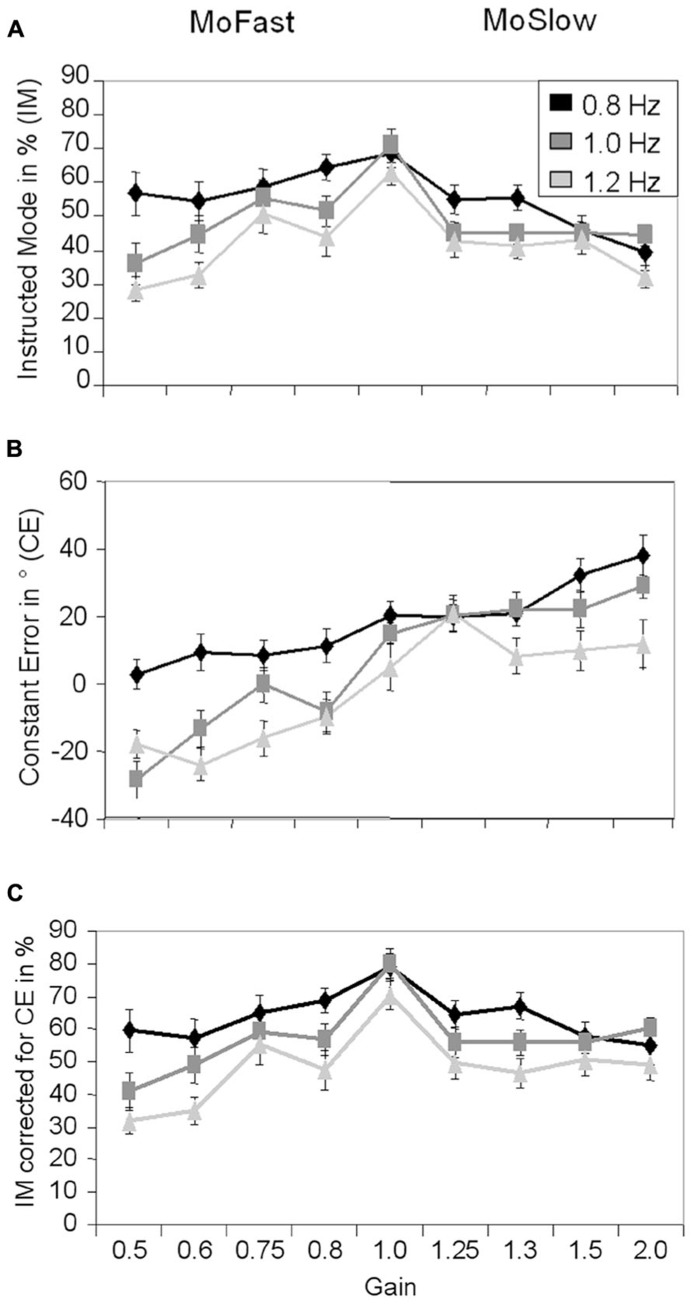
**Means and standard errors for Instructed Mode **(A)**, Constant Error **(B)**, and Instructed Mode corrected for Constant Error **(C)** depending on visual speed and gain.** MoFast = gains smaller than 1, movement speed is faster than visual speed. MoSlow = gains higher than 1, movement speed is slower than visual speed.

The results for IM are depicted in **Figure 2A**. A significant main effect of Visual Speed, *F*(2,26) = 27.20, *p* < 0.001, $\eta_{p}^{2}$ = 0.68, indicated that IM declined with increasing speed (0.8 Hz: *M* = 55.4%, 1.0 Hz: *M* = 48.7%, 1.2 Hz: *M* = 42.7%, *p*max = 0.005). A significant main effect of Gain, *F*(8,104) = 10.11, *p* < 0.001, $\eta_{p}^{2}$ = 0.44, indicated that IM was higher with gain = 1 (*M* = 67.7%) than in all other gains (*M*min = 40.4%, *M*max = 54.9%, *p*min < 0.001,* p*max = 0.026). A significant interaction between Gain and Visual Speed, *F*(16,208) = 2.76, *p* < 0.001, $\eta_{p}^{2}$ = 0.18, was also observed.

At 0.8 Hz speed, IM was significantly higher with MoFast gains (*M* = 58.6%) than MoSlow gains (*M* = 48.8%, *p* = 0.017). At the two faster speeds, IM did not significantly differ between MoSlow and MoFast gains (1.0 Hz: *p* = 0.62, 1.2 Hz: *p* = 0.52). Comparisons of the MoFast gains showed no significant differences in IM between gains at 0.8 Hz speed (*p*min = 0.15), but a significant decline in IM was observed between gain = 0.75 and 0.6 at 1.0 and 1.2 Hz speed (*p* = 0.016 and *p* = 0.002, respectively). The reverse was observed in MoSlow gains. Comparisons showed a decline in IM with higher gain at 0.8 Hz speed: IM was significantly lower with gain = 2 and gain = 1.5 than with gain = 1.3 and gain = 1.25 (*p* = 0.001), but the magnitude of gain did not significantly influence IM at 1.0 Hz (*p*min = 0.81) and 1.2 Hz speed (*p*min = 0.42).

### LEAD/LAG: CONSTANT ERROR

The results for CE are depicted in **Figure 2B**. A significant main effect of Visual Speed, *F*(2,26) = 45.13, *p* < 0.001, $\eta_{p}^{2}$ = 0.78, indicated that participants were more in advance/lagged less behind the stimulus with lower speed then with higher speed (0.8 Hz: *M* = 18.2°, 1.0 Hz: *M* = 6.5°, 1.2 Hz: *M* = -1.2°, *p*max = 0.002). A significant main effect of Gain, *F*(8,104) = 18.15, *p* < 0.001, $\eta_{p}^{2}$ = 0.58, indicated that participants lagged more behind/were less in advance of the stimulus with smaller gains than with larger gains. A significant interaction between Gain and Visual Speed, *F*(16,208) = 2.98, *p* < 0.001, $\eta_{p}^{2}$ = 0.19, was also observed. In MoFast gains CE was significantly more positive at 0.8 Hz speed (*M* = 8.1°) than at 1.0 Hz speed (*M* = -12.5°, *p* < 0.001) and 1.2 Hz speed (*M* = -16.8°, *p* < 0.001). CE did not significantly differ between the latter two speeds (*p* = 0.25). In MoSlow gains CE did not significantly differ between the 0.8 Hz (*M* = 27.8°) and 1.0 Hz speed (*M* = 23.4°, *p* = 0.15), but was significantly less positive at 1.2 Hz speed (*M* = 12.8°, *p*max = 0.005).

### CONTROL ANALYSES: IM CALCULATED FROM MEAN CE

One may argue that variations in IM are due to systematic variations in CE. Because IM was calculated by using CE values within ±45° around the ideal position, it may be that when the mean CE is not zero, parts of the distribution around it are systematically not used in the calculation of IM. To rule out this possibility, we recalculated IM, using a window around participants mean CE ± 45° for each condition. The results for IM corrected for mean CE are depicted in the **Figure 2C**. Results were similar to the original analysis of IM. Significant main effects of Gain, *F*(8,104) = 10.69, *p* < 0.001, $\eta_{p}^{2}$ = 0.45 and Visual Speed, *F*(2,26) = 44.96, *p* < 0.001, $\eta_{p}^{2}$ = 0.78, indicated that IM was highest with gain = 1 (*M* = 76.5%, *p*max = 0.008) and that IM decreased with increasing speed (0.8 Hz: *M* = 63.8%, 1.0 Hz: *M* = 57.0%, 1.2 Hz: *M* = 48.5%, *p*max = 0.004). A significant interaction between Gain and Visual Speed, *F*(16,208) = 2.06, *p* = 0.01, $\eta_{p}^{2}$ = 0.14, was also observed. In this analysis, IM did not significantly differ between MoFast and MoSlow gains at any speed (*p*min = 0.09). Comparisons between the MoFast gains showed again that the magnitude of gain did not significantly influence IM at 0.8 Hz speed (*p*min = 0.13), but a significant decline in IM was observed between gain = 0.75 and gain = 0.6 at 1.0 and 1.2 Hz speed (*p* = 0.02 and *p* = 0.001, respectively). Again, a different pattern was observed in MoSlow gains. Comparisons between the MoSlow gains showed a decline in IM with higher gain at 0.8 Hz speed, IM was significantly lower with gain = 2 and gain = 1.5 than with gain = 1.3 and gain = 1.25 (*p *= 0.004). No significant differences in IM were observed between gains at faster speeds (1.0 Hz: *p*min = 0.27, 1.2 Hz: *p*min = 0.54). Thus, negative and positive CE values did not obscure the general data pattern of IM.

### ANCHORING: VARIABLE ERROR

**FIGURE 3:**
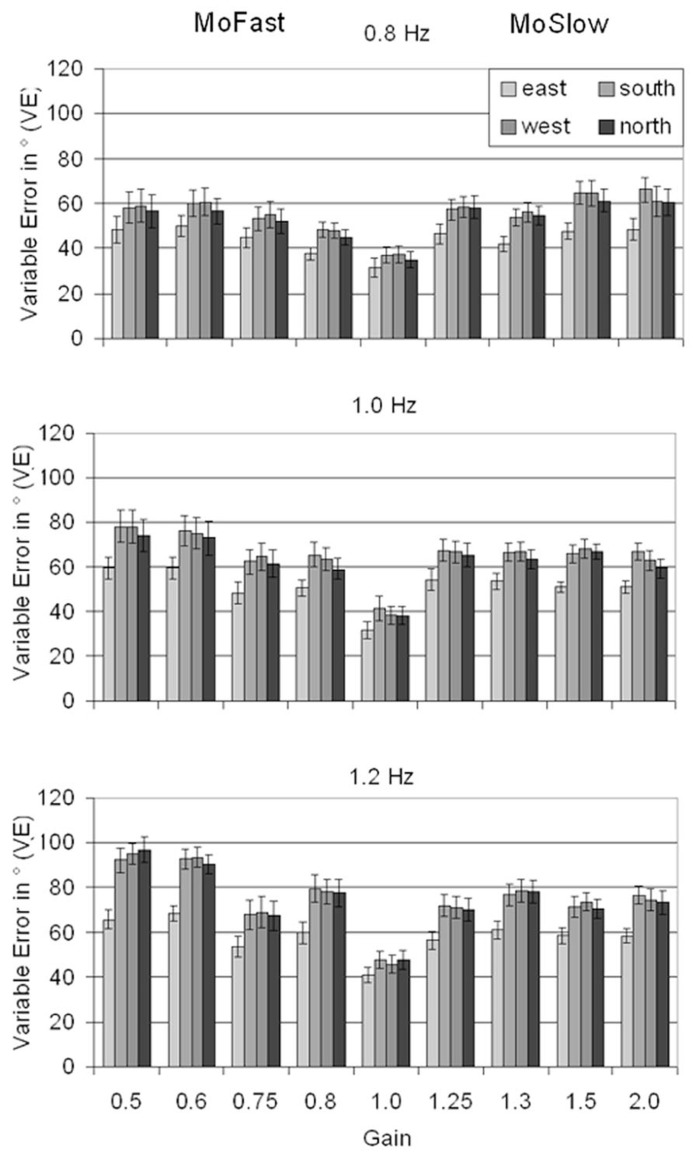
**Means and standard errors for Variable Error depending on location, gain, and visual speed.** MoFast = gains smaller than 1, movement speed is faster than visual speed. MoSlow = gains higher than 1, movement speed is slower than visual speed.

Results for VE are depicted in **Figure 3**. A significant main effect for Visual Speed, *F*(2,26) = 58.10, *p* < 0.001, $\eta_{p}^{2}$ = 0.82, showed that VE increased with increasing speed (0.8 Hz: *M* = 51.1°; 1 Hz: *M* = 61.0°; 1.2 Hz: *M* = 70.8°, *p*max < 0.001). A significant main effect of Gain, *F*(8,104) = 11.91, *p* < 0.001, $\eta_{p}^{2}$ = 0.48, indicated lower VE in gain = 1 (*M* = 39.3°) than in all other gains (*M*min = 58.3°, *M*max = 71.8°, *p*max < 0.001). The interaction between Gain and Visual Speed, *F*(16,208) = 2.08, *p* = 0.01, $\eta_{p}^{2}$ = 0.14, indicated that the increase in VE from 0.8 to 1.0 Hz was significantly larger in MoFast gains (*M* = 13.4°) than with gain = 1 (*M* = 2.4°, *p* = 0.013). Results were intransitive, the increase in MoSlow gains (*M* = 6.0°) did not differ significantly from the increase in MoFast gains (*p* = 0.04) and gain = 1 (*p* = 0.41). The increase in VE from 1.0 to 1.2 Hz did not significantly differ between MoFast gains (*M* = 12.4°), MoSlow gains (*M* = 7.7°), and gain = 1 (*M* = 7.9°, *p*min = 0.046)

Most importantly, a significant main effect of Location, *F*(3,39) = 164.35, *p* < 0.001, $\eta_{p}^{2}$ = 0.93, indicated that VE was lower when the stimulus dot was in the east (and, correspondingly, the effect dot in the west, *M* = 55.1°) than in the other locations (south: *M* = 65.4°; west: *M* = 65.3°; north: *M* = 63.4°, *p*max < 0.001). The significant interaction between Gain and Location, *F*(24,312) = 4.05, *p* < 0.001, $\eta_{p}^{2}$ = 0.24, reflected that the difference in VE between the east and the other locations was smaller for gain = 1 (*M* = 12.6°) than for all other gains (*M*min = 20.0°, *M*max = 38.0°, *p*max = 0.002). The interaction between Visual Speed and Location, *F*(6,78) = 9.94, *p* < 0.001, $\eta_{p}^{2}$ = 0.43, together with the significant interaction between Gain, Visual Speed, and Location *F*(48,624) = 2.55, *p* < 0.001, $\eta_{p}^{2}$ = 0.16, indicated that the difference between locations in VE increased with increasing speed in MoFast gains (differences east vs. other locations, 0.8 Hz: *M* = 9.2°, 1.0 Hz: *M* = 14.8°, 1.2 Hz: *M* = 21.4°, *p*max = 0.001), but no significant increase was found in gain = 1 (*p*min = 0.23) and MoSlow gains (*p*min = 0.12).

### AWARENESS OF THE TRANSFORMATION

**FIGURE 4:**
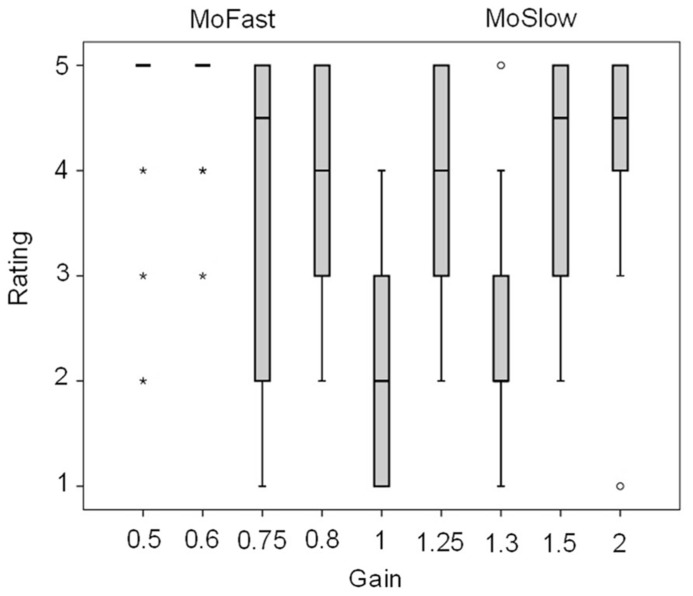
**Boxplots of the ratings of the presence of a transformation.** Mild outlier, between 1.5 and 3 × interquartile range above the third or below the first quartile, *extreme outlier, more than 3 × interquartile range above the third or below the first quartile. Verbal coding for ratings: 1 = certainly not present, 2 = likely not present, 3 = undecided, 4 = likely present, 5 = certainly present.

Box plots of the awareness ratings are displayed in **Figure 4**. Friedman’s test showed a significant effect of gain, χ^2^(8) = 48.7, *p* < 0.001. The presence of a transformation was less likely reported with gain = 1 than with other gains, apart from gain = 1.3 (*p* = 0.47, others: *p*min = 0.001, *p*max = 0.016). In MoFast gains the presence of a transformation was rated significantly more likely with gain = 0.5 and gain = 0.6 than with gain = 0.75 and gain = 0.8 (*p*min = 0.011, *p*max = 0.036). In MoSlow gains the presence of a transformation was rated less likely with gain = 1.3 than with all other gains (*p*min = 0.009, *p*max = 0.023), awareness ratings did not significantly differ between the other gains (*p*min = 0.21).

## DISCUSSION

In the present experiment we investigated how the perceptual-motor system deals with gain transformations in unimanual circling. Participants were instructed to coordinate a visual feedback dot of their hand movement with a continuously circling stimulus dot in order to produce mirror symmetric circular movements of the two dots on the screen. The movement angle of the hand was multiplied by a gain factor before being presented on the screen. We used four gains smaller than 1 (MoFast), a gain of 1, and 4 gains larger than 1 (MoSlow). Speed of the feedback dot was varied in three levels. Accuracy of performance (IM) was highest with gain = 1. Accuracy declined with increasing speed. In MoFast gains the magnitude of gain did not matter in slow speed but performance declined in lower gains with increasing speed. In MoSlow gains accuracy declined in higher gains with slow speed, but not with faster speed. The analysis of CE showed that participants were more likely to lag behind the stimulus with higher speed than with lower speed. Further, with small gains participants lagged behind the stimulus, whereas with higher gains participants were in lead of the stimulus. Because systematic variations in CE may cause variations in IM, we recalculated IM corrected for mean CE. The data pattern remained the same, showing that CE did not compromise the original IM analysis. Participants showed anchoring in the middle of the screen where the two circles were closest to each other (east location of the stimulus dot, west location of the feedback dot). The difference between the east and the other locations was smaller for gain = 1 and increased less with speed in gain = 1 and MoSlow gains than MoFast gains.

The data show that the mere presence of an angular gain transformation affects coordination in unimanual circling negatively. Performance with regular feedback (gain = 1) was more accurate than performance with gains larger or smaller than 1. Thus, the same (perceptually easy) visual pattern was harder to produce if a transformation was present. If only the visual pattern had mattered for performance, the different gains between hand movement and feedback should have had no effect on performance. Difficulty of the task did also not depend on movement speed in a simple manner, because then a decline in performance from large to small gains should have been observed. Rather, the results are in favor of the assumption that the presence of a transformation affects performance negatively. This is in accordance with results showing that the transformation itself is an important part of the cognitive representation of tool-use actions (Massen and Prinz, 2007; Lepper et al., 2008). The results are in contrast to studies in which straight movements were investigated: here either accuracy decreases with increasing gain, or higher gains are compensated for with longer movement durations (Rosenbaum and Gregory, 2002; Rieger et al., 2005; Sutter et al., 2008). An explanation is that introducing a gain in circling movements implies a constant change in the mapping of hand position to feedback position, which is not the case in straight movements. It seems that with a constant mapping change limitations in performance do not (only) depend on a speed-accuracy relationship. Rather, there may be flaws in the representation of the transformation, resulting in an increased difficulty to predict the movement’s consequences in external space (see below).

It was further of interest whether the magnitude of the transformation or merely its presence matters for performance. This depended on speed. In MoFast gains the magnitude of gain did not matter with slow speed but performance declined in lower gains with increasing speed. The effect of transformation magnitude in the MoFast gains with higher speed may be due to movement speed: coordination may be more difficult with faster speed due to higher demands on the motor system. This is corroborated by the finding that accuracy generally declined with increasing speed (see also Kelso, 1984; Haken et al., 1985; Heuer, 1993; Byblow et al., 1995; Carson et al., 1997; Roerdink et al., 2005). A different picture was apparent in MoSlow gains: accuracy declined in higher gains with slow speed, but not with faster speed. How can this be explained? It could be that slow movements with high gain are difficult because of the slowness of the hand movements; participants may have preferred to move faster. Studies have shown that there is a preferred movement speed for continuous movements, which also influences how movements at other speeds are performed (Naruse et al., 2001). This interpretation is corroborated by the results on the CE, which indicated that participants were more in lead of the stimulus with higher gains and slower speed.

The CE was systematically influenced by the magnitude of the transformation and visual speed. With lower speed and higher gain participants were more in lead of the stimulus, with higher speed and lower gain participants lagged behind the stimulus. With gain = 1 and in MoSlow gains participants were slightly in lead of the stimulus. The tendency that overall feedback was more likely to be in lead of the stimulus may be due to participants’ use of the dominant hand in the task, as the dominant hand shows a slight lead over the non-dominant hand when coordinating symmetrical movements in bimanual coordination (Treffner and Turvey, 1995). However, as this effect seems to be due to attentional rather than motoric factors (the lead of the dominant hand disappears when attention is directed to the non-dominant hand, Amazeen et al., 1997), an alternative explanation is that participants paid more attention to the feedback than the stimulus. The data pattern also suggests that the CE is related to movement speed: higher visual speed (and correspondingly movement speed) resulted in more lag/less lead. Similar, lower gain, also implying higher movement speed, resulted in more lag/less lead.

It is assumed that the nervous system controls movements using internal models (Wolpert and Flanagan, 2001), with inverse models choosing appropriate motor commands for desired goals and forward models predicting the sensory consequences of motor commands. The predictions can refer to bodily consequences (the hand movement itself) but also to the movement consequences in external space, like visual feedback. External consequences do not necessarily coincide with the bodily consequences when the movement is transformed as in tool use (Wolpert and Flanagan, 2001). In tool use people develop internal models of the tool transformation (Imamizu et al., 2000, 2003, 2007; Verwey and Heuer, 2007; Rieger et al., 2008; Sülzenbrück and Heuer, 2009, 2012). In the present task internal models need to take the gain transformation into account. Our data suggest that this may be insufficiently accomplished: with high gains/low movement speed the feedback resulting from a movement might be underestimated, resulting in the feedback being in advance of the transformation. Conversely, with small gains/high movement speed, the feedback produced by the movement may be overestimated, resulting in the feedback lagging behind the stimulus. This is in accordance with findings that the nervous system does not necessarily completely adapt to observed errors (Wei and Kording, 2009). Thus, there seem to be flaws in the representation of the transformation.

We also investigated whether participants show visual anchoring, i.e., reduced variability at salient locations of the dots’ trajectories. Anchoring occurred where the two trajectories were closest to each other (east position of the stimulus dot and west position of the feedback dot). Because the position of the hand could not be determined by the position of the feedback dot in the present experiment, except with gain = 1, the actual hand position was not relevant for anchoring to occur in this position. Larger and smaller differences between the east and the other locations in variability (smaller difference for gain = 1 than other gains, higher difference with higher visual speed in MoFast gains) can be explained by overall task performance. Conditions in which variability was lower also showed lower differences between the east and the other locations. Importantly, the data show that for anchoring to occur, discrete timing events like tones (Fink et al., 2000; Keller and Repp, 2008), or movement reversals/maximal excursions (cf. Roerdink et al., 2008) are not necessary. Rather, visually salient locations are sufficient. They may serve a similar function as such events.

Circle drawing usually results in equal temporal variability along the entire trajectory (Spencer and Zelaznik, 2003). Therefore, circle drawing tasks are thought to require emergent timing in contrast to other tasks like tapping to a metronome which require event-based timing (Zelaznik et al., 2002). In contrast to our study previous results indicate that anchoring does not occur in circle drawing even when participants are asked to produce one circle between two beats of a metronome (Studenka and Zelaznik, 2011). However, when participants are not drawing freely, but place the stylus inside a circular track, anchoring at the timing target seems to occur (Repp and Steinman, 2010). The use of a crank for in the present task may thus have contributed to the occurrence of anchoring.

We argued that anchoring occurs when the circles are in the position closest to each other. An alternative explanation is that rather than the visual proximity of stimulus and feedback, the leftmost position of the circle produces the effect. Being in the leftmost position of a circle may have perceptual advantages over being at other position of a circle. However, the comparison locations we chose were at points for which similar arguments could be made (rightmost, topmost, and lowermost). Nevertheless, such an effect might also explain the differences in results between previous studies: Repp and Steinman (2010) used the west position of the circles for synchronization and found anchoring with a metronome, whereas Studenka and Zelaznik (2011) used the north and found no anchoring. As we did not vary the closest position between stimulus and feedback in our experiment, this has to remain an open question for future studies.

The magnitude of gain had an impact on participants’ awareness of the transformation in MoFast gains. The greater the gain diverged from gain 1, the more likely participants noticed the presence of a transformation in MoFast gains. In MoSlow gains this effect was also apparent but less clear (the presence of a transformation was rated more likely with gain = 1.25 than gain = 1.3, only the latter one was rated less likely than the higher gains). The observation that the magnitude of the gain mattered for awareness of the transformation is interesting: one could have expected that due to the constant change of the mapping of movement positions to feedback position with any gain other than 1 a transformation would always be detected equally well. Even with small deviations in gain from 1 there are eventually circles in which movement and feedback are on opposite sides. The results are in accordance with studies indicating that participants are not very good in knowing their actual hand positions in similar tasks and that the magnitude of a perturbation plays an important role for detecting it (Fourneret and Jeannerod, 1998; Knoblich and Kircher, 2004; Sutter et al., 2008; Müsseler and Sutter, 2009). During visible movements, proprioception does not seem to be attended to (Proteau and Isabelle, 2002), and processing of proprioceptive feedback may be masked by processing of visual feedback (Tremblay and Proteau, 1998). The observation, that even with gain = 1 participants were not sure that no transformation was presented, corroborates the interpretation that participants’ awareness of the actual hand position may have been limited. Thus, the magnitude of the transformation may be more important for detecting it than a mismatch between movement and feedback position. Nevertheless, the observation that participants were not sure that no gain was present with gain = 1 may also be due to the design of the experiment: the presence of a transformation was more likely than its absence, which may have led participants to believe that a transformation was always present.

The present results have implications for the use of tools with gain transformations, which involve a constant change in the mapping of movement positions to feedback positions. First, such movements are more difficult to perform than untransformed movements. Thus, there are limits to the dominance of visual feedback in controlling actions involving tool transformations (see also Sutter et al., 2013). Second, the representation of the transformation in internal models can be flawed. It is important to note, that the performance decrements and flaws in the representation of the transformation were observed even though the initial adaptation phases to gains and speeds were excluded from the data analysis. It could however be, that with extended practice further adaptation processes take place.

In conclusion, the size of an angular gain transformation as well as its mere presence influence performance in a situation in which the mapping of movement positions to visual feedback positions is not constant. The representation of angular gain transformations by internal models may be flawed. Anchoring (reduced variability) at visually salient locations supports the coordination of transformed feedback with external events. Participants’ conscious experience of the transformation depends on its magnitude. When designing machines or tools that involve transformations between movements and their external consequences, one should be aware that the mere presence of angular gains may result in performance decrements.

## Conflict of Interest Statement

The authors declare that the research was conducted in the absence of any commercial or financial relationships that could be construed as a potential conflict of interest.
